# Protective effect of paeoniflorin against oxidative stress in human retinal pigment epithelium in vitro

**Published:** 2011-12-29

**Authors:** Xie Wankun, Yu Wenzhen, Zhao Min, Zhou Weiyan, Chen Huan, Du Wei, Huang Lvzhen, Yongsheng Xu, Li Xiaoxin

**Affiliations:** Department of Ophthalmology, People’s Eye Institute, Peking University People’s Hospital, Key Laboratory of Vision Loss and Restoration, Ministry of Education, Beijing, China

## Abstract

**Purpose:**

This study was conducted to determine whether paeoniflorin (PF) could prevent H_2_O_2_-induced oxidative stress in ARPE-19 cells and to elucidate the molecular pathways involved in this protection.

**Methods:**

Cultured ARPE-19 cells were subjected to oxidative stress with H_2_O_2_ in the presence and absence of PF. The preventive effective of PF on reactive oxygen species (ROS) production and retinal pigment epithelium (RPE) cell death induced by H_2_O_2_ was determined by 2’,7’- dichlorodihydroﬂuorescein diacetate (H_2_DCFDA) fluorescence and 3-(4, 5-dimethylthiazol-2-yl)-2, 5 diphenyl tetrazolium bromide (MTT) assay. The ability of PF to protect RPE cells against ROS-mediated apoptosis was assessed by caspase-3 activity and 4', 6-diamidino-2-phenylindole (DAPI) staining. Furthermore, the protective effect of PF via the mitogen-activated protein kinase (MAPK) pathway was determined by western blot analysis.

**Results:**

PF protected ARPE-19 cells from H_2_O_2_-induced cell death with low toxicity. H_2_O_2_-induced oxidative stress increased ROS production and caspase-3 activity, which was significantly inhibited by PF in a dose-dependent manner. Pretreatment with PF attenuated H_2_O_2_-induced p38MAPK and extracellular signal regulated kinase (ERK) phosphorylation in human RPE cells, which contributed to cell viability in ARPE-19 cells.

**Conclusions:**

This is the first report to show that PF can protect ARPE-19 cells from the cellular apoptosis induced by oxidative stress. The results of this study open new avenues for the use of PF in treatment of ocular diseases, such as age-related macular degeneration (AMD), where oxidative stress plays a major role in disease pathogenesis.

## Introduction

Age-related macular degeneration (AMD) is the leading cause of irreversible vision loss in the developed world among persons older than 50 years of age [1,2]. AMD progresses through two stages (early and advanced). Clinically and histologically the retinal pigment epithelium (RPE) is thought to be the prime early target for the disease. Early AMD is characterized by changes in the pigmentation of the RPE and an accumulation of extracellular deposits between RPE cells and Bruch’s membrane [3]. The alterations finally result in RPE cell death, subsequent atrophy of the photoreceptors, and loss of vision. As a result, an approach to rescue RPE cells would be helpful for preventing the occurrence or progression of AMD.

Growing evidence supports an essential role for oxidative stress in the development of age-related RPE cell dysfunction [4]. Oxidative damage is likely to be higher in cells that have a high metabolic rate, such as RPE cells. The retina–RPE exists in an environment that is rich in endogenous sources of reactive oxygen species (ROS). Due to an imbalance between the generation and the elimination of ROS, RPE cells are damaged by cumulative oxidation [5]. Transient fluctuations of ROS could serve some regulatory function, whereas high and sustained levels of ROS cause mitochondrial DNA damage and ultimately leads to the apoptosis of RPE cells [6]. Oxidative stress is also known to activate mitogen-activated protein kinases (MAPKs), which include stress-activated p38 mitogen-activated protein kinase (p38MAPK), extracellular signal-regulated kinase (ERK), and c-Jun N-terminal kinase (JNK). The MAPK pathway is one of the most ubiquitous signal transduction systems and plays an important role in the apoptosis and proliferation of RPE cells [7,8]. It is becoming increasingly clear that early AMD treatment should focus on rescuing RPE cells from oxidative damage. Several epidemiologic studies show that increased dietary intake and supplementation with specific antioxidant nutrients may reduce the risk for AMD [9,10].

Paeoniflorin (PF), a monoterpene glucoside, is known to be one of the principal active components of Paeonia Radix, a traditional Chinese herbal medicine derived from the root of *Paeonia lactiflora* Pall (family Ranunculaceae), which is traditionally used for the treatment of eye disorders [11–13]. This compound has been reported to have various pharmacological activities, such as antioxidant, anti-inflammatory, and neuroprotective effects on various types of cells [14–16]. PF is also known as a heat shock protein-inducing compound and shows cell-protective activities against varies form of stress [17,18]. Despite these compelling observations, the mechanism by which PF protects RPE cells from oxidative stress is not completely understood.

The purpose of this study was to investigate the effects of PF on quiescent and oxidative-stressed RPE cells in vitro and to discover the possible mechanisms involved in the ROS and MAPK pathways. We used the well characterized model of H_2_O_2_-induced oxidative stress in ARPE-19 cells as the in vitro model system. We showed for the first time that PF can protect human RPE cells from ROS-induced apoptosis through the MAPK signal pathway.

## Methods

### Cell culture and drug preparation

Human RPE cells (ARPE-19 cell line) were obtained from the American Tissue Culture Collection (Manassas, VA) and were cultured in Dulbecco's modified Eagle's medium (DMEM)/F-12 human amniotic membrane nutrient mixture (DMEM/F12; Sigma Aldrich, St. Louis, MO) with 10% fetal bovine serum (FBS; Invitrogen-Gibco, Grand Island, NY), 100 U/ml penicillin, and 100 μg/ml streptomycin at 37 °C under 5% CO_2_ and 95% humidified air and were used at passage 10–15 as we previously described [19].

To maintain ARPE-19 cells in an undifferentiated state, cells were passaged before obtaining confluence. To obtain differentiated cells, cells were grown to confluence and then maintained in DMEM-F12 medium with serum reduced to 1% for another 2 weeks [20]. These cultures reach confluence 2–3 weeks after passaging and differentiate within 4–6 weeks; the cultures can be kept in a differentiated state for extended culture periods. Both the undifferentiated and the differentiated ARPE-19 cells were maintained and passaged in basal media. They were plated in the densities mentioned in the description of each assay and incubated overnight; the cells were then incubated with fresh medium for an additional 24 h and were starved in serum free-DMEM/F12 medium for 24 h. The cells were then washed and treated with the appropriate concentration of drugs in DMEM/F12 medium.

PF (purity>99%) was purchased from the China National Institute for Control of Pharmaceutical and Biologic Products (Beijing, China). PF was dissolved in DMEM/F12 medium without FBS supplement to a stock concentration of 2 mg/ml and diluted to appropriate concentrations in culture media before use.

### Cell viability assay

The 3-(4, 5-dimethylthiazol-2-yl)-2, 5 diphenyltetrazolium bromide (MTT) assay was used to determine cell viability. Briefly, differentiated and undifferentiated ARPE-19 cells grown in 96-well plates were washed twice with phosphate-buffered saline (PBS; pH 7.4, with components: potassium phosphate monobasic, 0.001 M; sodium chloride, 0.155 M and sodium phosphate dibasic, 0.003 M) and then starved in DMEM/F12 medium overnight before treatment. Cells were incubated with different concentrations of H_2_O_2_ and PF (5–200 μg/ml) in medium containing 1% FBS for 24 h. The control medium was 1% FBS. The medium was then replaced with fresh medium containing 0.5 mg/ml MTT for 4 h at 37 °C. After incubation, the medium was carefully removed from the plate, and 200 μl dimethyl sulfoxide was added to resolve the formazan crystals. The optical density (OD) was measured with an enzyme-linked immunosorbent assay (ELISA) plate reader (Dynatech Medica, Guernsey, UK) at an emission wavelength of 570 nm. Background-subtracted OD values were normalized with the drug-free control and expressed as viability percentages. Each experiment was performed in three wells and was duplicated at least three times.

### Isolation of total RNA and RT–PCR

Total RNA was isolated (Trizol; Invitrogen, Carlsbad, CA) according to the manufacturer’s instructions and was quantified by spectrophotometry. Reverse transcription was then performed by using 100 ng of RNA and the First-Strand cDNA Synthesis kit (Promega, Madison, WI). PCR was performed with Taq PCR Master Mix kit (Qiagen, Hilden, Germany) according to the manufacturer’s instructions for 35 cycles. Each cycle consisted of 30 s at 94 °C, 30 s at 55 °C and 45 s at 74 °C. Specific primers used for retinal pigment epithelium 65 (*RPE65*) were forward 5′-AAC CTC TTC CAT CAC ATC AAC ACC-3′ and reverse 5′-GAT TCA AGC CAA GTC CAT ACG C-3′. Primers for fibroblast growth factor receptor-1 (*FGFR-1*) were forward 5′-CGG CAG CAT CAA CCA CAC ATA C-3′ and reverse 5′-AGC ACC TCC ATC TCT TTG TCG G-3′. Experiments were performed in triplicate and repeated three times.

### 4,6-diamidino-2-phenolindole staining

4,6-diamidino-2-phenolindole (DAPI; Sigma Aldrich, St. Louis, MO) staining was conducted for the identification of apoptotic nuclei as previously described [21]. ARPE-19 cells (1×10^5^ cells) were treated with 200 μM H_2_O_2_ and PF in various concentrations. Cells were fixed and stained with 10 μg/ml of DAPI. After incubation for 5 min in the dark, the cells were washed, mounted on slides, and observed under a fluorescence microscope (BX50; Olympus, Tokyo, Japan). Experiments were performed in triplicate and repeated three times.

### Intracellular reactive oxygen species measurement

Intracellular ROS measurement assay was performed as previously described [22]. Briefly, ARPE-19 cells (1×10^4^ cells/well) in 96-well plates were washed with PBS and incubated for 20 min at 37 °C in PBS containing 20 μM 2,7-dichlorodihydrofluorescein diacetate (Sigma Aldrich). The cells were washed with PBS, incubated with 200 μM H_2_O_2_ in PBS for 2 h at 37 °C, and washed twice with PBS to remove the H_2_O_2_ from the wells. After these washes, the cells were incubated with PF at different concentrations in PBS for 2 h at 37 °C and washed with PBS to remove the PF from the wells. Intracellular ROS production was measured on a spectrofluorometer (Gemini EM microplate; Molecular Devices Corporation, Sunnyvale, CA) with an excitation of 485 nm and emission of 530 nm. Nonspecific fluorescence values without cells were subtracted from the fluorescence values with cells. The experiments were performed in triplicate and repeated three times.

### Caspase-3 activity measurement

Caspase-3 activity was determined with a caspase-3 cellular activity assay kit (Merck, Darmstadt, Germany) according to the manufacturer’s protocol. In this assay, the capacity of cellular caspase-3 to cleave the labeled substrate DEVD-p-nitroaniline (DEVD-pNA) was measured spectrophotometrically. Apoptosis was induced by incubation with H_2_O_2_. ARPE-19 cells (2×10^5^ cells) were incubated with 200 μM H_2_O_2_ or 200 μg/ml PF for 2 h. The cells were harvested and then suspended in the cell lysis buffer. Cell lysates were incubated in the presence or absence of 5 μl DEVD-pNA for 1 h at 37 °C. Absorbance was measured at 405 nm in a microplate reader (Versa-Max; Molecular Devices). Uninduced and induced cells without substrate served as the background control. Induced cells were incubated with DEVD-CHO, an inhibitor of caspase-3, as a negative control. Experiments were performed in triplicate and repeated three times.

### Western blot analysis

Western blot analysis was performed using standard western blotting methods as we have previously described [23]. Cells were washed twice with ice-cold PBS, lysed on ice with buffer (RIPA; 50 mM Tris-HCl [pH 7.4], 150 mM NaCl, 1 mM EDTA, 1% NP-40, 0.5% sodium deoxycholate) containing a protease and phosphatase inhibitor cocktail (Sigma Aldrich). Cell lysates were then sedimented in a microfuge at 15,000× g for 10 min. The supernatant was collected, and the protein content of each lysate was measured by using a BCA protein assay kit (Pierce, Rockford, IL) according to the manufacturer’s instructions. Equal amounts of protein were separated by electrophoresis on 10% SDS–PAGE and were transferred electrophoretically on nitrocellulose membranes (Amersham, LittleChalfont, UK). After blocking nonspecific binding with 5% skim milk, the membranes were incubated with a rabbit polyclonal antibody against p38MAPK (1:1,000), ERK1/2 (1:1,000), JNK (1:1,000), phosphorylated forms of p38MAPK (1:1,000), ERK1/2 (1:2,000) or JNK (1:1,000; Cell Signaling Technology, Danvers, MA), followed by incubation with a horseradish peroxidase–conjugated goat antibody against rabbit immunoglobulin (IgG; 1:2,000; Cell Signaling Technology, Danvers, MA). The signals were visualized (ECL kit; GE Healthcare Life Sciences, Buckinghamshire, UK) according to the manufacturer’s protocol. For sequential blotting with additional antibodies, the membranes were stripped with a restored western blot stripping buffer and reprobed with the indicated antibodies. Western blot analyses were repeated three times, and qualitatively similar results were obtained.

### Statistical analysis

Statistical differences between groups were evaluated using the Student paired *t* test after the normal distribution test. All statistical tests were performed with SPSS for Windows, version 13.0 (SPSS, Chicago, IL). A value of p≤0.05 was considered statistically significant.

## Results

**Figure 1:**
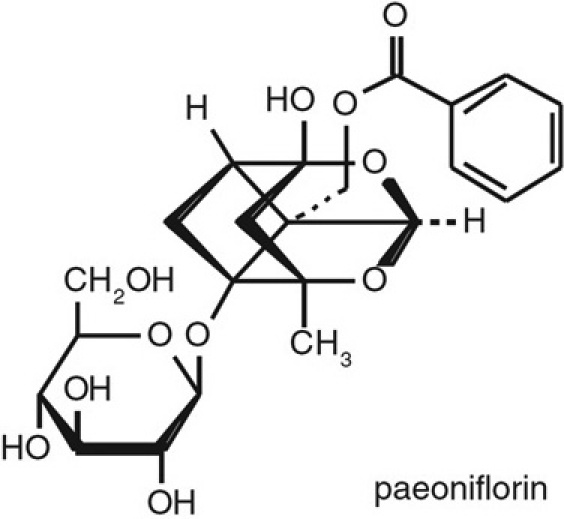
Chemical Structure of Paeoniflorin (PF), C_23_H_2_8O_11_, molecular weight 480.45.

In this study, we tested the effectiveness of PF (Figure 1) in protecting RPE cells from oxidative stress.

### Protection of paeoinflroin from H_2_O_2_-induced cell death in RPE cells

**Figure 2:**
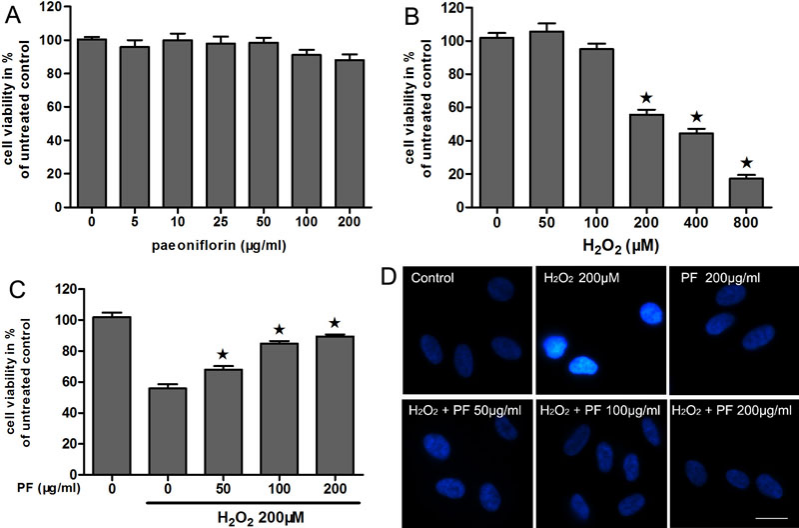
Paeoniflorin (PF) protects human retinal pigment epithelium cells from H_2_O_2_-induced cell death. **A**: Cytotoxicity of paeoniflorin to ARPE-19 cells were measured by 3-(4, 5-dimethylthiazol-2-yl)-2, 5 diphenyl tetrazolium bromide (MTT) assay. ARPE-19 cells were treated with or without different concentrations of PF (5–200 μg/ml) for 24 h, and cell viability was assessed by an MTT assay. The results are expressed as percentage of control, and each value represents the mean±SEM of three independent experiments (n=3 experiments). **B**: Cell viability of ARPE-19 cells following H_2_O_2_ exposure were measured by MTT assay. The cells were treated with or without different concentrations of H_2_O_2_ (50–800 μM) for 24 h. Cell viability was measured by an MTT assay. The results are expressed as percentage of control, and each value represents the mean±SEM of three independent experiments (n=3 experiments, *p<0.05). **C**: Cell viability was measured in ARPE-19 cells treated with 200 μM H_2_O_2_ or different concentrations of PF (50–200 μg/ml) by an MTT assay. The results are expressed as percentage of control, and each value represents the mean±SEM of three independent experiments (n=3 experiments, *p<0.05). **D**: Cell viability was measured using 4', 6-diamidino-2-phenylindole (DAPI) staining. Strong fluorescent spots show apoptotic nuclei. Figures were selected as representative data from three independent experiments. Scale bar, 10 μm.

To investigate the cytotoxicity of PF on human RPE cells, we performed a cell viability assay. As shown in Figure 2A, PF did not significantly affect the cell viability of ARPE-19 cells up to 200 μg/ml. The data indicate that PF is relatively safe for ARPE-19 cells.

H_2_O_2_ has been used in the design of models of oxidative stress in RPE cells by many investigators because of its rapid membrane permeability and because it is a well established model to investigate the mechanism of oxidative stress in RPE cells [24–27]. For these reasons, we selected H_2_O_2_ for our studies and performed a series of dose–response assays to determine the working concentrations that led to a consistent and high degree of cytotoxicity, which we defined as the level of H_2_O_2_ that killed 50% of the RPE cells after a 24-h incubation.

We initially used low-density proliferating cultures of RPE cells because of their high sensitivity to oxidative stress [22]. As we expected, the viability of ARPE-19 cells decreased in a H_2_O_2_ dose-dependent manner (Figure 2B). We found that 200 μM H_2_O_2_ caused cell viability to decrease by about half (56%), and reliable and consistent cytotoxicity was obtained in undifferentiated ARPE-19 cells.

To evaluate the cytoprotective effect of PF on H_2_O_2_-induced cell death, we investigated whether H_2_O_2_-treated cell viability of human RPE cells could be prevented by PF treatment. The MTT assay showed that cell viability of ARPE-19 cells was significantly decreased after treatment with 200 μM H_2_O_2_. However, PF treatment effectively prevented ARPE-19 cells from H_2_O_2_-induced cell death in a dose-dependent manner (Figure 2C). The difference in cell survival rate under 200 μM H_2_O_2_ with and without PF was significant (p<0.05). These data indicate that PF effectively protects ARPE-19 cells from H_2_O_2_-induced cell death in a dose-dependent manner.

To evaluate the effects of oxidative stress on cell growth, we monitored the changes of APRE-19 cells in morphology and apoptosis after exposure to 200 μM H_2_O_2_. After 24-h exposure, nuclear staining of the cells revealed strong fluorescent spot and pyknotic nuclei, indicating condensed chromatin and apoptosis bodies. However, the strong fluorescent spot was almost completely abrogated by pretreatment with PF (Figure 2D).

### Protective effect of paeoniflorin on differentiated retinal pigment epithelium cells

Because RPE cells are differentiated in vivo and are likely to be present at various densities depending on the health of the retina, we decided to compare the responses of the undifferentiated and differentiated cells to determine whether the state of differentiation influenced the protective effect of PF.

**Figure 3:**
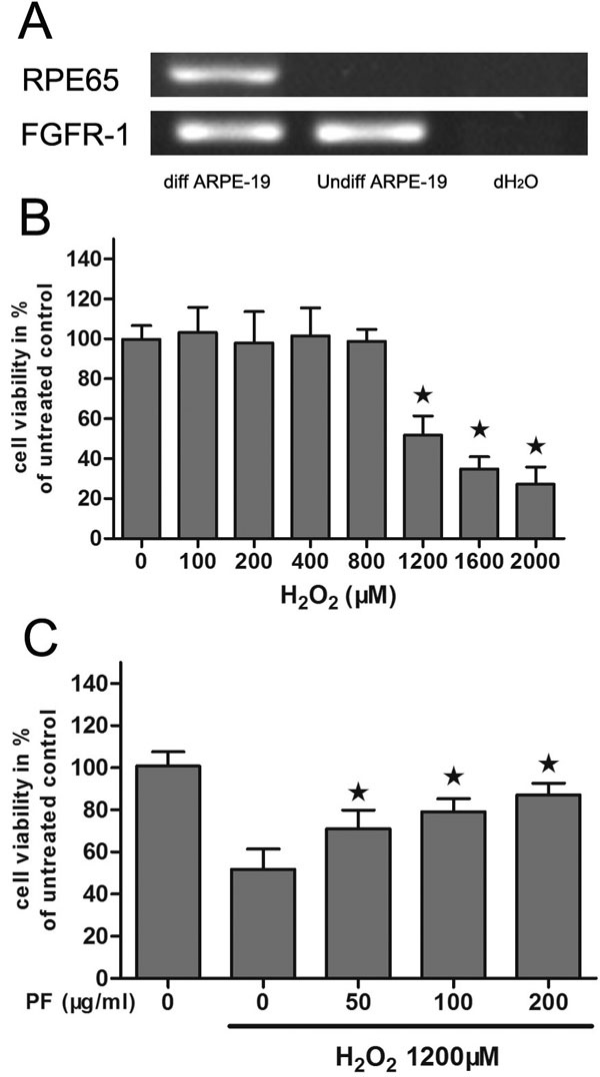
Protective effects of paeoniflorin (PF) on H_2_O_2_-induced differentiated ARPE-19 cells. **A**: Levels of retinal pigment epithelium 65 (RPE65) and fibroblast growth factor receptor-1 (FGFR-1) mRNA were measured by Reverse transcriptase PCR in undifferentiated (Undiff ARPE-19) and differentiated (Diff ARPE-19) ARPE-19 cells. Total RNA from undifferentiated or differentiated ARPE-19 cells was reverse transcribed and amplified using primers for RPE65 and FGFR1. A reaction omitting the cDNA was used as a negative control (NC). **B**: Cell viability of differentiated ARPE-19 cells following H_2_O_2_ exposure were measured by MTT assay. The cells were treated with or without different concentrations of H_2_O_2_ (100–2,000 μM) for 24 h. Cell viability was measured by an MTT assay. The results are expressed as percentage of control, and each value represents the mean±SEM of three independent experiments (n=3 experiments, *p<0.05). **C**: Cell viability was measured in differentiated ARPE-19 cells treated with 1,200 μM H_2_O_2_ or different concentrations of PF (50–200 μg/ml) by an MTT assay. The results are expressed as percentage of control, and each value represents the mean±SEM of three independent experiments (n=3 experiments, *p<0.05).

Most of our early studies were performed on undifferentiated ARPE-19 cells, which are quite sensitive to oxidative stress. To obtain differentiated cells, cells were grown to confluence and then maintained in DMEM-F12 medium with the serum reduced to 1% for 2 weeks. To investigate the expression of a cellular marker of differentiated RPE cells, the expression of *RPE65* was evaluated by reverse transcriptase (RT)-PCR analysis. As shown in Figure 3A, *RPE65* was highly expressed in the differentiated cells.

Since differentiated RPE cells are highly resistant to oxidative stress, we first investigated the cytotoxicity of H_2_O_2_ on differentiated RPE cells to establish the appropriate concentration for the studies. As shown in Figure 3B, cell viability was not significantly affected by 800 μM H_2_O_2_, while 1,200 μM H_2_O_2_ caused cell viability to decrease by about half (52%). This concentration was therefore used to evaluate the cytoprotective effect of PF on differentiated cells.

Next, the MTT assay showed that PF treatment also effectively prevented differentiated RPE cells from H_2_O_2_-induced cell death in a dose-dependent manner (Figure 3C). This was similar to the data shown in Figure 2C in which relatively undifferentiated ARPE-19 cells were plated at lower cell densities. The results showed that the cytoprotective effect of PF was independent of the state of ARPE-19 cell differentiation.

### Paeoniflorin inhibits H_2_O_2_-induced reactive oxygen species production in human retinal pigment epithelium cells

To determine whether the cytoprotective effect of PF is a consequence of an intracellular effect or whether it is simply due to ROS scavenging activity in the extracellular media, we investigated whether PF could be transported into RPE cells to inhibit H_2_O_2_-induced intracellular radical production, using cell permeable fluorescence dye. Briefly, cells were first stressed with H_2_O_2_ before removing the H_2_O_2_ from the cells; PF was then added for 2 h before intracellular ROS levels were measured. In this way, because there is no extracellular contact between the PF and the H_2_O_2_, any reduction in ROS levels could be attributed to an intracellular effect.

**Figure 4:**
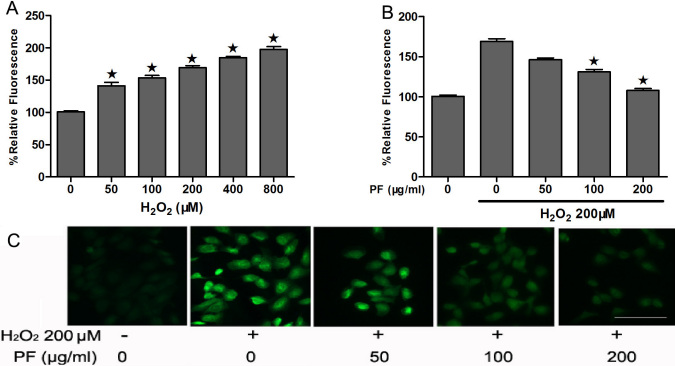
Paeoniflorin (PF) inhibits H_2_O_2_-induced reactive oxygen species (ROS) production in ARPE-19 cells. **A**: APRE-19 cells were treated with different concentrations of H_2_O_2_ (50–800 μM). For measuring H_2_O_2_ production, the cells were then labeled with 2’,7’-dichlorodihydroﬂuorescein diacetate (H_2_DCFDA). Quantitative analysis was performed by measuring fluorescence intensity. Each value represents the mean±SEM of three independent experiments (n=3 experiments, *p<0.05). **B**: ARPE-19 cells were treated with 200 μM H_2_O_2_ and different concentrations of PF (50–200 μg/ml). For measuring H_2_O_2_ production, the cells were then labeled with H_2_DCFDA. Quantitative analysis was performed by measuring the fluorescence intensity relative to the control. Each value represents the mean±SEM of three independent experiments (n=3 experiments, *p<0.05). **C**: For measuring H_2_O_2_ production, the cells were labeled with H_2_DCFDA. Figures were selected as representative data from three independent experiments. Scale bar, 100 μm.

As demonstrated in Figure 4A, treatment of ARPE-19 cells with H_2_O_2_ led to an increase in intracellular ROS levels compared with untreated cells. The intensity of the mean oxidized DCF peak was increased by 1.6 fold compared with controls after 200 μM H_2_O_2_ treatment in ARPE-19 cells. However, PF attenuated the ROS accumulation in a dose-dependent manner (Figure 4B). The difference in ROS production under 200 μM H_2_O_2_ with and without PF was significant (p<0.05). This observation indicates that PF inhibits H_2_O_2_-induced ROS production in a dose-dependent manner.

### Paeoniflorin inbibits H_2_O_2_-induced apoptosis through suppression of caspase-3 activity

**Figure 5:**
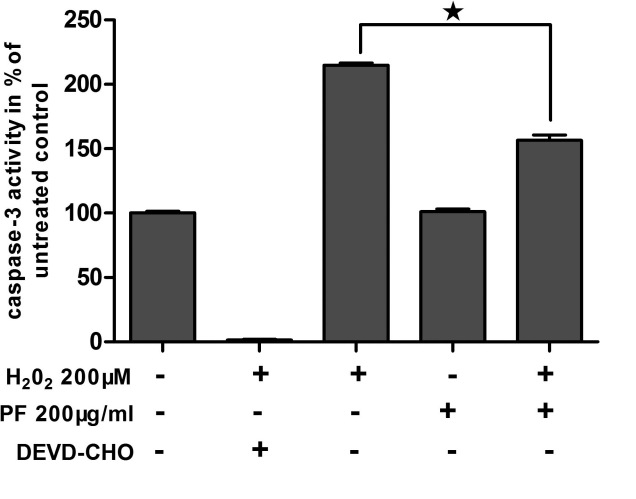
Paeoniflorin (PF) protects ARPE-19 cells from H_2_O_2_-induced apoptosis via inhibition of caspase-3 activity. ARPE-19 cells were treated with 200 μM H_2_O_2_ or 200 μg/ml PF. Caspase-3 protease activity in ARPE-19 was determined as the release of p-NA from the substrate and was monitored colorimetrically at 405 nm. Quantitative analysis was performed by measuring the fluorescence intensity relative to the untreated cells. A negative control-induced sample was incubated with caspase-3 inhibitor (DEVD-CHO) before the addition of substrate. Each value represents the mean±SEM of three independent experiments (n=3 experiments, *p<0.05).

To investigate the mechanism by which PF protects human RPE cells from oxidative stress, the inhibitory effect of PF on caspase-3 activity related to apoptotic processes was assessed. As demonstrated in Figure 4, treatment of RPE cells with 200 μM H_2_O_2_ for 2 h caused an elevation of caspase-3 (2.1 fold). However, the increased activity of caspase-3 was significantly inhibited by pre-incubation with 200 μg/ml PF. The difference in the increase of caspase-3 activity of cells treated with PF and H_2_O_2_ and of cells treated with H_2_O_2_ alone was statistically significant (p<0.05). Incubation with 200 μg/ml PF alone for 12 h did not show any changes in caspase-3 activity (Figure 5).

### H_2_O_2_-induced mitogen-activated protein kinase phosphorylation

**Figure 6:**
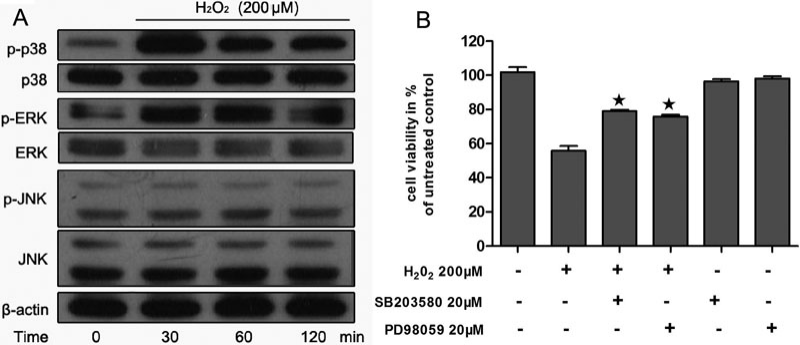
Mitogen-activated protein kinase (MAPK) were activated by H_2_O_2_ in ARPE-19 cells. **A**: Human RPE cells were exposed to 200 μM H_2_O_2_ for various time points (30, 60, 120 min) and cell lysates were prepared. The phosphorylated and total protein levels of p38 mitogen-activated protein kinase (p38MAPK), extracellular signal regulated kinase (ERK), and c-Jun N-terminal kinase (JNK) were detected with specific antibodies, using western blot analysis as described in the Methods. β-actin served as the loading control. Figures were selected as representative data from three independent experiments. **B**: After 24-h exposure to H_2_O_2_, cell viability was measured with an MTT assay. Each value represents the mean±SEM of three independent experiments (n=3 experiments, *p<0.05).

H_2_O_2_-mediated cell death in RPE cells involves the activation of stress kinases [28]. To examine the kinetics of MAPK activation, cells were exposed to 200 μM H_2_O_2_ for various times. Phospho-p38MAPK levels increased to a maximum with 30 min and subsequently slowly declined. Phospho-ERK1/2 levels increased to a maximum with 60 min and lasted for 120 min. Phospho-JNK levels remain unchanged in 120 min. No change in the levels of total MAPKs and β-actin was seen (Figure 6A). These observations indicate that H_2_O_2_ can induce p38MAPK and ERK activation in a time-dependent manner.

To determine whether these MAPKs are related to cell death, activation of p38MAPK and ERK was inhibited by relative kinase inhibitors and cell viabilities were determined by MTT assay. As show in Figure 6B, inhibitors of p38MAPK (20 μM SB203580) and ERK (20 μM PD98059) partly prevented the loss of cell viability of H_2_O_2_-induced RPE cells. This suggests that blocking of p38MAPK and ERK activation to some extent increased cell viability of H_2_O_2_-induced RPE cells.

### Paeoniflorin inhibits H_2_O_2_-induced mitogen-activated protein kinase activation

**Figure 7:**
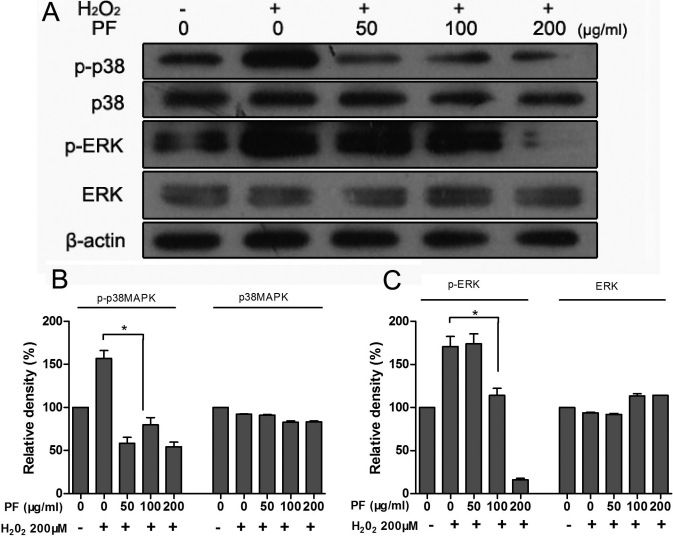
Paeoniflorin (PF) inhibits H_2_O_2_-induced MAPK activation in ARPE-19 cells. ARPE-19 cells were pretreated with different concentrations of PF for 4 h and exposed to 200 μM H_2_O_2_ for 30 min; cell lysates were then prepared. The phosphorylated and total protein levels of p38MAPK and ERK1/2 were detected with specific antibodies, using western blot analysis as described in the Methods. β-actin served as the loading control. Figures were selected as representative data from three independent experiments. Quantitative analysis was performed by measuring the intensity relative to the control. Each value represents the mean±SEM of three independent experiments (n=3 experiments,*p<0.05).

To investigate whether the cytoprotective effect of PF on APRE-19 cells is related to the MAPK signal pathway, western blot analysis on phospho-p38MAPK and phospho-ERK after treatment with 200 μM H_2_O_2_ and different concentrations of paeoniflorin was performed. As show in Figure 7, protein levels of phospho-p38MAPK and phospho-ERK in cells treated with PF (50–200 μg/ml) before H_2_O_2_ exposure decreased compared to that of cells exposed to H_2_O_2_ only. Total MAPKs and β-actin levels were not greatly affected by this treatment. This suggests that PF partly inhibited the activation of p38MAPK and ERK.

## Discussion

The present study reveals, for the first time to our knowledge, the protective effect of PF on oxidative injury in human RPE cells, together with the underlying molecular mechanisms. In our study PF protected human RPE cells from oxidative stress-induced death with good efficacy, high potency, and low toxicity.

PF, isolated from *P. lactiflora*, is one of the major constituents of peony plants. Peony plants were widely used for many centuries in traditional Chinese medicine for the treatment of eye disorders [11–13]. Peony extracts and their constituents have been shown to have various biologic and biomodulating activities, including improvement of memory, antioxidant activity, and anti-inflammatory activity [14–16]. Therefore, PF is well known as a scavenger of ROS in extracellular environments and for its cytoprotective effect on other cell types against the oxidative damage of 60Co-ray irradiation [14]. However, whether PF possesses similar activity in RPE cells against the damage of H_2_O_2_-induced oxidation is not known. In the present study, we evaluated the protective activity of PF against H_2_O_2_-induced injury in RPE cells. We first demonstrated that PF could decrease H_2_O_2_-induced cell death, caspase-3 activity, and MAPK activation in RPE cells. These effects will benefit the prevention of AMD.

Regarding the possible mechanisms by which PF inhibits H_2_O_2_-induced RPE cell death, our results showed that PF may act through antioxidative effects. It has been proposed that oxidative stress may be a key contributor to RPE dysfunction and degeneration in the pathology of AMD. We selected H_2_O_2_ as an oxidative inducer in the model of oxidative stress in RPE cells because H_2_O_2_ has rapid membrane permeability and depolarizing effects on the mitochondrial membrane potential and has been used by many investigators [21,22,29]. H_2_O_2_ is a direct oxidant that is formed by the RPE cell under normal physiologic conditions and must be neutralized on a continual basis [22,30].

We performed our initial studies with undifferentiated ARPE-19 cells because of their high sensitivity to oxidative stress. However, in vivo RPE cells are differentiated and grow as a monolayer, which can withstand oxidative stress; we therefore investigated oxidative stress on differentiated RPE cells.

We first evaluated the expression of RPE65 by RT–PCR analysis to identify the cell differentiation phenotype and found that RPE65 was highly expressed on differentiated ARPE-19 cells. We then investigated the cytotoxicity of H_2_O_2_ on differentiated cells and found that the concentration of H_2_O_2_ necessary to kill 50% of the differentiated cells was higher than for undifferentiated cells.

Our findings are consistent with other investigators who reported a similar density-dependent cytotoxicity of H_2_O_2_ in primary RPE and ARPE-19 cells [27,31–33]. These researchers suggested that the amount of oxidant per cell but not the concentration of H_2_O_2_ influences oxidative toxicity [33]. Thus, consistent with our results, to reach the same amount of oxidant per cell, differentiated ARPE-19 cells at a high density require higher concentrations of H_2_O_2_ oxidants [33].

These data appear to be contradictory to those obtained in the previous study by Bailey and coworkers [27], which showed that ARPE-19 cells grown in monolayers can withstand H_2_O_2_ concentrations as high as 5 mM with little effect on cell viability when treated for 24 h. One of the main differences between our experimental paradigm and that of Bailey et al. [27] was that we included a period of serum withdrawal before oxidative stress. Bailey et al. [27] also showed that serum withdrawal significantly reduced viability in the 5-week cultures. Our results are similar to previous research by Alizadeh and coworkers [34], which showed that the viability of differentiated ARPE-19 cells after treatment with H_2_O_2_ was unchanged up to doses of 300 μM but decreased to 55% at 1 mM and to 31% at 3 mM. These results suggest that there is a synergistic effect between serum withdrawal and H_2_O_2_ treatment.

PF can effectively rescue H_2_O_2_-induced RPE cell death in a dose-dependent manner in both differentiated and undifferentiated cells, implying that the cytoprotective effect of PF was independent of the state of ARPE-19 cell differentiation.

We have shown that H_2_O_2_ strongly enhances intracellular ROS production in human RPE cells and that this was significantly inhibited by PF treatment. Furthermore, we have also shown that PF can inhibit caspase-3 activity to protect RPE cells from apoptosis [14]. Caspase-3 is a frequently activated death protease, catalyzing the specific cleavage of many key cellular proteins in the process of apoptosis. In accordance with these data in human RPE cells, caspase-3 activity after oxidative stress was similarly markedly diminished by PF. It is possible that the inhibition of intracellular ROS production and caspase-3 activity by PF is causally related to the prevention of H_2_O_2_-induced cell death.

The MAPK pathway links the extracellular survival signal to the apoptosis-related pathway and plays a critical role in keeping cells alive by blocking the apoptotic pathway. We thus assessed the roles of the MAPK pathway and H_2_O_2_-induced modification of the MAPK-signaling pathway in oxidative injury. In our study, H_2_O_2_ activated p38 MAPK and ERK1/2 in RPE cells. Several studies have shown activation of MAPK by oxidative stress in the RPE. However, results from these studies are controversial. Ho et al. [28] showed that JNK and p38MAPK were activated during H_2_O_2_ stimulation and that H_2_O_2_-induced cell death was significantly reduced by their specific inhibitor. Qin et al. [35] reported an activation of all three main MAPK pathways in H_2_O_2_-induced RPE cells, but inhibition of ERK, JNK, and p38MAPK with pharmacologic inhibitors did not enhance or rescue H_2_O_2_-induced cell death. Glotin et al. reported that exposure of RPE cells to t-BHP only activates the MEK/ERK1/2, but neither JNK nor p38MAPKs were involved, and specifically blocking ERK1/2 activation protects against oxidative stress-induced cell death [36]. Alge-Priglinge et al. [29] showed that H_2_O_2_-induced an upregulation of phosphorylated ERK1/2 and p38MAPK but the levels of phosphorylated JNK remained unchanged. In the present study there is an evident upregulation of phosphorylated ERK1/2 and p38 MAPK when RPE cells are exposed to H_2_O_2_ but the levels of phosphorylated JNK remain unchanged. This result is in accordance with Alge-Priglinge et al. PF significantly attenuated H_2_O_2_-induced activation of p38MAPK and ERK1/2 in a dose-dependent manner. Thus, the cytoprotective effect of PF may be associated with the inhibition of H_2_O_2_-induced oxidative stress-mediated activation of these MAPK pathways in this in vitro model.

We found it interesting that ERK1/2, which has been reported to regulate cell survival [7], was activated by H_2_O_2_ and paeoniflorin reduced its activation. This reduction in ERK1/2 activation correlated to a reduction in H_2_O_2_-induced cell death. This finding seemingly contradicted a potential cell survival role for ERK1/2; however, this observation may be explained by the broad antioxidant activity of PF. Clearly, the regulation of cell survival and death through the MAPK pathway is dependent on several factors, such as the state of cell confluence, serum starvation, or type, dose, and duration of oxidative stimulus. Thus, the regulation of apoptosis by MAPKs is more complex than initially thought. Collectively, our findings support a protective role of PF in APRE-19 cells exposed to an H_2_O_2_ challenge through the inhibition of p38MAPK and ERK1/2 activation.

PF, as a heat shock protein (HSP) inducer, had an enhancing effect on HSP induction by heat shock and can induce an effect on HSP expression [17]. HSP plays a role in protecting cells from environmental deleterious stresses. Molecular chaperones are able to inhibit the aggregation of partially denatured proteins and refold them, as demonstrated by in vitro and in vivo studies [37]. Hsp27 expression has been shown to protect cells from death cascades induced by oxidative stress [38]. The mechanism of HSP in the protective effect of PF on H_2_O_2_-induced RPE damage should be further investigated. Thus, further work will focus on this important question to determine the contribution of HSP on the protective effect of PF on H_2_O_2_-induced damage of RPE.

Most of the current model systems to study RPE oxidative stress use acute and high doses of chemical treatment, such as H_2_O_2_. While AMD is a long-standing process occurring in vivo, it is likely that oxidative stress in AMD is chronic and below levels that cause RPE cell death in vivo is chronic and below levels that cause RPE cell death. Also, death of RPE cells occurs only at a later stage in pathological AMD [39]. If we could elucidate the protective effects of PF under such severe cell damage conditions (cell death), then PF should have the protective potential to be even more effective under milder cell damage conditions that only affect phagocytosis and lysosomal functions.

Although there are some limitations to the study of AMD in vitro, such as the dose of oxidant used in acute studies might not be relevant to the in vivo condition, and AMD is a variously progressive disease, H_2_O_2_-induced RPE cell death is a widely used model to investigate the mechanism of oxidant-induced RPE cell death [21,29,40,41]. We chose H_2_O_2_ to induce RPE cell death in our study for several reasons. First, H_2_O_2_ has been used by other investigators and its effects on RPE cells have been well characterized. H_2_O_2_ has been reported to trigger apoptosis in RPE cells and the initial loss of RPE cells in AMD result from apoptosis. Second, exogenous H_2_O_2_ could enter the cells and induce cytotoxicity because of its high membrane permeability. Third, H_2_O_2_ could induce a high degree of oxidative stress. If we could elucidate the protective effects of PF under such high degree of oxidative stress, then PF should have the protective potential to be even more effective under milder, more physiologic conditions of stress.

ARPE-19 is an adult human RPE cell line that displays many differentiated properties of the RPE in vivo and is widely used as an in vitro model for investigation of molecular pathways and related cellular functions of human RPE [41,42]. Since the physiologic function of RPE cells is responsible for clearance of oxidants derived from photoreceptor turnover, ARPE-19 cells have been used to explore oxidant-mediated insults and related protective factors in various studies [21,22,25,40,41]. However, ARPE-19 cells are not identical to RPE cells in vivo. Also, the results from in vitro studies may not fully reflect the in vivo progress of PF transport in the retina. Further studies to investigate the effects of PF on RPE under in vivo conditions are warranted.

In summary, the present study showed that PF could protect human PRE cells against H_2_O_2_-induced oxidative stress, as measured by cell viability, apoptosis, caspase-3 activity, ROS activity, and the MAPK-signaling pathway. PF-mediated protections can be conferred by one or more of the following mechanisms: first, PF could inhibit the production of ROS, which could induce RPE cell death; second, PF could suppress caspase-3 activity, which is a crucial mediator of apoptosis; third, PF could inhibit the phosphorylation of p38MAPK and ERK, which are important signaling pathways in H_2_0_2_-induced RPE cell death. Therefore, PF may be an attractive candidate in preventing the slow visual impairment caused by AMD.
